# Why Reviewing Apps Is Not Enough: Transparency for Trust (T4T) Principles of Responsible Health App Marketplaces

**DOI:** 10.2196/12390

**Published:** 2019-05-02

**Authors:** Til Wykes, Stephen Schueller

**Affiliations:** 1 King's College London Department of Psychology Institute of Psychiatry, Psychology and Neuroscience London United Kingdom; 2 Center for Behavioral Intervention Technologies North Western University Chicago, IL United States

**Keywords:** mobile health, digital health applications, consumer protection, ehealth, advertising standards, digital mental health interventions, digital health

## Abstract

The overselling of health apps that may provide little benefit and even harm needs the health community’s immediate attention. With little formal regulation, a light-touch approach to consumer protection is now warranted to give customers a modicum of information to help them choose from the vast array of *so-called* health apps. We suggest 4 guiding principles that should be adopted to provide the consumer with information that can guide their choice at the point of download. We call these the Transparency for Trust (T4T) principles, which are derived from experimental studies, systematic reviews, and reports of patient concerns. The T4T principles are (1) privacy and data security, (2) development characteristics, (3) feasibility data, and (4) benefits. All our questions are in a simple form so that all consumers can understand them. We suggest that app stores should take responsibility for providing this information and store it with any app marketed as a health app. Even the absence of information would provide consumers with some understanding and fuel their choice. This would also provide some commercial impetus for app developers to consider this requested information from the outset.

## Background

Digital therapeutics are being touted as having the potential to transform health care by improving people’s experience, increasing effectiveness, and reducing costs. A few digital aids are recommended and integral to health services, but much of the e-health field depends on overselling [1]. This business plan seems to be working as the digital health field was estimated to be worth $25 billion globally in 2017 [2,3]. One US survey found that 58% of smartphone users have downloaded at least one health app [4]. The overselling of health apps needs the health community’s immediate attention as many of these apps may provide little benefit and some apps may cause harm. With little formal regulation, alternative light-touch approaches to consumer protection are necessary to give customers a modicum of information as a basis for choosing from the vast array of *so-called* health apps.

We believe that simple, but informative, evidence should be available at the point of downloading and propose 4 succinct aspects that represent the critical information required for responsible health app marketplaces. We refer to these 4 principles—privacy and data security, development practices, feasibility, and health benefits—as the Transparency for Trust (T4T) principles. The goal of these principles is to operationalize efforts that can be made by app marketplaces to answer calls for better oversight and backing up products with data and research [3,5,6]. These T4T principles draw from several sources including patient and regulatory perspectives, recent systematic reviews, and experimental studies (eg [4,7-14]). We have used the definition of a health app from Innovate UK as those apps that contribute to the physical, mental, or social well-being of the user [15]. Our principles are designed to be applicable to the whole panoply of health apps from sleep apps to diabetes apps, symptom trackers to mindfulness interventions eg[16-18]. The fastest growing segment of digital therapeutics is health apps. Current estimates suggest that over 300,000 health apps exist with a recent yearly growth rate of 25% [17]. The rapid growth of products and lack of growth of information and regulation have resulted in very little information to separate quality health apps from those that are at best useless and at worst harmful. As a result, consumers are left to navigate the app stores alone. For one fast-growing sector, digital mental health, the likelihood of using an app is also affected by the relative lack of access or choice of mental health services as well as the stigma and discrimination experienced by sufferers and the interrelationship with physical health. We know, for instance, that people with depression face a higher risk of developing heart disease than individuals without depression and that following a heart attack, each additional depressive symptom that develops increases the risk of another heart attack by 15% [17]. Mental health problems also affect morbidity in other disorders such as rheumatoid arthritis and asthma [16]. We have used examples from mental health, but our principles are intended to inform and empower all health app users.

## Why Do We Need Some Simple Principles?

### Exponential Growth and Poor Regulation

The use of digital technologies to alleviate, prevent, or maintain health has been recognized for many years, but although it offers enormous potential to rethink how services are provided, there are large roadblocks in its way (eg [19]). A Lancet Psychiatry Commission suggested that digital therapeutics could provide benefits now to complement current mental health treatments and aid self-management [8]. However, evidence suggests that some apps are not only ineffective, unsafe, and hard to use, but do not meet users’ privacy and security expectations [9,20,21].

Formal regulation is remarkably light and restricted to a narrow selection of health apps that provide formal diagnoses or treatment for specified medical conditions [3,22,23]. Even when an app is regulated, we cannot be sure that it will work. For example, the US Food and Drug Administration (FDA) recently approved the first behavioral health app, reSET, for the treatment of substance use by using evidence from a clinical trial of a Web-based version of the treatment, not the app itself [24]. In the United Kingdom, the Care Quality Commission issued guidance in 2017 for digital health care providers, but this concentrated on safety [25]. The Medicines and Healthcare Products Regulatory Agency (MHRA) provides Conformité Européenne marking for medical devices and Certificates of Free Sale [26] but leaves review to the National Information Board. As it stands, many health apps are marketed with few checks and even regulatory approval appears to offer little confidence on whether that specific product was ever directly evaluated.

More complex regulation has been proposed. The National Institute of Health and Clinical Excellence (NICE) is curating an NHS National Health Service app library. This is a burdensome process, and so few apps will be assessed in any year. Currently, the library has 78 apps, with only 18 for the fastest developing sector, mental health. This is a minute subset of the 325,000 available [27]. In the United States, the FDA launched a precertification pilot program involving 9 companies to speed the approval process, but this will evaluate the developers and their practices rather than focusing on the product [28]. Apple Inc has introduced additional requirements for medical apps for developers, but these focus mainly on measurement accuracy [29]. There is a middle way to fill this important, and now yawning, gap in consumer information. Health app marketplaces could take a lead by providing relatively simple guidance.

## What is Wrong With Current Systems for Reviewing Health Apps?

Most proposed evaluations (eg, Mobile App Rating Scale, MARS [30], Enlight assessment tools [31]) assess usability, aesthetics, content, user engagement, and available research evidence, and others have been adding to this list [32]. These systems are useful because they facilitate multifaceted and thorough evaluations of apps, but they fall short of allowing clear recommendations. In fact, more recent evidence from Canada involving service users demonstrated that a high MARS rating would not on its own provide enough information to allow service users to form a decision on whether to download an app [33]. Advisory bodies such as NICE in the United Kingdom make a determination of what is likely to be cost-effective (effective, cost relative to benefit, and other comparison treatments), before they recommend its use in the UK NHS. Consumers, however, want to make choices based on simpler information. One for-profit company, ORCHA [34], provides reviews based on *current standards, regulation and good practice*, but their overall score does not allow a consumer to decide which components are important to them. PsyberGuide [35], a nonprofit organization, also provides reviews that include a service user focus but does not receive data directly from app developers. But apart from their lack of fulfilling all users’ expectations, no method provides clear information at the point of sale, and a potential consumer would have to search in 2 places for the information they need, to make a choice. We propose only 4 aspects of apps that represent the critical information required for responsible health app marketplaces. These 4 principles, deemed the T4T principles, are privacy and data security, development characteristics, feasibility data, and benefits.

## Transparency for Trust (T4T) Principles

### Privacy and Data Security

Privacy and data security are a primary concern for patients and their clinicians [36-38], and its importance has only become more salient with recent events such as the Facebook and Cambridge Analytica scandal [39-41]. The European Union General Data Protection Regulation is strong and introduces new rights for people to access the information companies hold about them, obligations for better data management for businesses, and a new regime of fines across Europe. There are weaker regulations elsewhere, resulting in varying protections internationally. One review prompted the closure of the NHS app store when it was discovered that accredited apps were not encrypting data adequately and did not explicitly describe the personal data leaving the app [42]. Happtique, an early app certification company, met a similar fate when several of its certified apps were hacked, demonstrating the inadequacy of its processes to evaluate privacy and data security [43,44]. Many apps rely on selling the data they collect for their business plan, which jeopardizes personal privacy [42]. There is also evidence of poor practice resulting in fines for selling sensitive information to Lottery companies and fraudsters [45]. Privacy concerns change with the evolving technology, even though device operating systems are moving toward encryption on the device by default. Nevertheless, users need information about data leaving the app to make informed decisions about their willingness to provide sensitive health information [46,47].

Although full formal audits are needed to ensure apps follow their stated procedures [48], even requiring developers to list their privacy and data security procedures in simple terms would be a significant step forward on raising standards [49]. We propose 3 questions: (1) what data leave the device? (2) how are those data stored? (eg, de-identified, encrypted), and (3) who will have access to those data? It should be clear what, if any, data are being sold, to whom, and what steps are taken to ensure that users cannot be identified by those data.

### Development Characteristics

Development characteristics describe how the app was developed, and our recommendations conceptually overlap with those of the FDA’s precertification pilot and the MHRA in the United Kingdom. Good developmental practices would involve all stakeholders (clinicians and the target audience) as well as using evidence-based guidance from the beginning and at all stages of development and testing. The absence of the use of guidance or standards has recently been noticed for physical activity and fitness apps where very few of the thousands of Android apps provided any measurement or used any of the accepted guidance [50]. We especially emphasize including the target audience. This may seem obvious, but unfortunately, development practices often include clinicians and experts but more rarely involve the target audience until evaluation. Many studies rely on small numbers of participants or convenience samples, for example, soliciting feedback from stressed college students rather than individuals with depression [51]. Again, this may seem obvious, but independent usability evaluations have demonstrated that many popular commercial apps are frustrating and challenging for members of the intended audience, raising questions about their prior involvement and the potential for the app to benefit this community [52]. Recent evidence also suggests that good design contributes not only to usability but also engagement with health apps [53], and there are several authoritative descriptions of the processes for developing good design [54].

Developers should outline their design and development process and clearly describe how patients were involved. Our 3 questions are as follows: (1) how were target users involved in the initial design? (2) how were target users involved in usability evaluations? and (3) has usability been independently evaluated?

### Feasibility

Feasibility evaluations should address how people use the app (usability and user experience), how long they use it (engagement), and whether any serious adverse concerns are discovered (safety). These aspects provide information on how people use the app, including expectations on the frequency and length of use. This information is also vital to assess benefits. It would not be possible to run a drug trial or market a drug without some concept of the dosing frequency and expected therapeutic dose, and the same should be true with health apps.

Again, we have 3 questions: (1) what proportion of users continue to use the app after 2 weeks? (2) what adverse events occurred and what was the rate of those events? and (3) has feasibility been independently evaluated? We propose a 2-week test not because it represents a likely therapeutic dose but because very few users persist in using a health app after the first week [55]. A standard metric, such as 2 weeks, could promote cross-app comparisons in engagement. Like usability testing, independent evaluation of apps is the key to promote transparency and confidence in findings. Despite the availability of engagement analytics, few are reported even in the clinical assessments of feasibility [56]. Independent evaluations could be carried out by service user groups, which could further strengthen service user involvement in the process of development and evaluation. Transparency could be further facilitated by making these datasets available to the research community.

### Health Benefits

Health benefits are apparent from rigorous evaluations using standardized and accepted outcomes for the target condition that provides an indication of health benefits. Although many researchers have noted the mismatch between the development cycle for mobile apps and traditional randomized controlled trials [57,58], it is still the case that health apps presented as digital therapeutics require rigorous evaluation to back up their claims. The speed of development should not preclude such evaluations as suggested by some academics and designers [59]. We should be presented with direct evidence on an app’s safety and effectiveness because they are not merely mobile versions of websites even if they have similar content. People use apps differently, including more frequently and in shorter bursts [60], and these differences could affect their impact. We have already mentioned that this is happening with the FDA-approved (and first) behavioral health app, reSET, using clinical trial evidence from a Web-based version of the treatment [32]. Although triangulation of different sorts of data has been suggested (eg, MindTech [61]), we believe that health apps should undergo a trial to determine their superiority to other treatment options, especially as many unsubstantiated claims have been made [36]. Advertising standards require evidence to support any claims made, so these data fulfill both commercial and patient needs. Evaluations should also consider opportunity cost as using a health app may delay treatments that could be more beneficial, or a delay could worsen the health condition, making it harder to treat. All these benefits and costs need to be weighed in the balance. Our 3 questions are: (1) what is the impact on the health condition? (2) what percentage of users received either no benefit or deteriorated? and (3) are there specific benefits that outweigh any costs?

## What Would This Look Like in Practice?

We have inserted the information from 4 health apps, one of which was named the app of the year in the iTunes Store in 2017 (Calm) (see Table 1). We have extracted, where possible, the information on each of our principles from the information provided with the app. The differences are very clear, especially in privacy and health benefits. There could, of course, be more data available on benefits held elsewhere, but this was not available at the point of download. However, what these simple principles also provide is the ability for a consumer to trade off the attributes. Some may want to know that their data are totally secure, whereas others might want to allow some encrypted anonymized data to be transmitted if the effectiveness of the app is proven. Indeed, in a recent survey of participants recruited from a mood and anxiety disorder clinic, many respondents were willing to allow an app to collect data directly from one’s phone, including global positioning system motion sensors, and screen state [37].

**Table 1:** Evaluating apps with the Transparency for Trust principles.

Transparency for Trust principle		Apps			
		BlueIce^a^	Calm^b^	My Fitness Pal^c^	Dario Diabetes Management^d^
**Privacy and security**					
	1) What data leave the device?	No information leaves the device.	Device identifiers, user settings, device operating system, use of app, and location.	Data related to lifestyle (eg, sleeping habits), life events, dietary restrictions, fitness goals, height, weight, measurements, fitness level, heart rate, sleep data, body mass index, biometric data, similar types of data relating to physiological condition and activity, and personal data (name, email address, postal code, date of birth, and contact number).	Personal information (registration information, such as full name, gender, email address, phone number, and birth date; financial information, such as PayPal account or credit card number; voluntary information; health information, such as diabetes type; and device information) and nonpersonal information (nonidentifiable information such as software and hardware information).
	2) How are those data stored?	All data are stored on the app and owned by the user.	No information.	All data are stored on the company’s server	All data are stored on the company’s server.
	3) Who will have access to those data?	Only user of the device where BlueIce is installed has access.	Third party: service providers, marketers with Calm, other systems (Google Fit of HealthKit,), industry research, etc.	Partners and affiliates, service providers and vendors, and social network providers.	Unspecified third parties.
**Development characteristics**					
	1) How were target users involved in the initial design of the app?	Coproduced by Oxford Health NHS Foundation Trust and young people with lived experience.	No information.	User testing on various app iterations, but no additional information provided.	User testing on various app iterations, but no additional information provided.
	2) How were target users involved in usability evaluations?	Not provided.	Not provided.	User testing on various app iterations, but no additional information provided.	User testing on various app iterations, but no additional information provided.
	3) Has usability been independently evaluated?	No independent usability evaluations were conducted.	Independently evaluated by PsyberGuide.org.	No independent usability evaluations were conducted.	Independently evaluated by Orcha.com; Food and Drug Administration approval.
**Feasibility**					
	1) What proportion of users continue to use the app after 2 weeks?	93% of users kept using it.	No information provided.	No information provided.	No information provided.
	2) What adverse events occurred in the test population and what was the rate of those events?	None found (clinicians did not withdraw user and users did not feel app use increased self-harm).	No information provided.	No information provided.	No information provided.
	3) Has feasibility been independently evaluated?	No independent evaluations conducted.	No independent evaluations conducted.	No information provided.	No information provided.
**Benefits**					
	1) What was the impact on clinical outcomes?	Significant reductions in depression and anxiety, and 73% reduced self-harm after 12 weeks.	No clinical outcomes research reported.	No clinical outcomes research reported.	No clinical outcomes research reported.
	2) What percentage of users received no benefit or deteriorated?	27% reported no reductions in self-harm.	No description provided of nonresponders or users who deteriorated.	No description provided of nonresponders or users who deteriorated.	No description provided of nonresponders or users who deteriorated.
	3) Are the specific benefits worth the cost?	No information provided about the expected ratio of benefits to risks.	No information provided about the expected ratio of benefits to risks.	No information provided about the expected ratio of benefits to risks.	No information provided about the expected ratio of benefits to risks.

^a^BlueIce is a prescribed evidence-based app to help young people manage their emotions and to reduce urges to self-harm [62].

^b^Calm was the 2017 app of the year on the Apple store and is for meditation and sleep [63].

^c^My Fitness Pal is for logging and motivating physical activity and diet and was the top-rated app in the journal *Men’s Health* in 2018 [64].

^d^Dario Diabetes Management monitors blood glucose history, allows carbohydrate counting, and will send messages to up to 4 people if your levels. Scored relatively highly by third-party app rater, Orcha [65].

## Are These Principles Different From Those Suggested by Others?

As we have said, T4T principles were based on those suggested by others in recent years. However, we more clearly operationalize our principles into concrete questions that could be answered and made available to potential users. To do so, we considered information important to regulators, developers, and health services, as well as integrating patient viewpoints taken from a number of different studies [37,66]. Patients are, after all, the consumer group of interest. Their views do not necessarily coincide with the expert groups’ views, as shown in the Delphi exercise by Zelmer et al [33]. Privacy and security feature in every assessment system and in regulations and are high on the list for patients, especially those with a mental health problem who may be more sensitive about information about them being shared [11]; So, it is included here, but in the simplest terms and not buried in an incomprehensible privacy statement. Our principle for a *fit-for-purpose* app includes development with patients. This principle is often suggested [19] but rarely incorporated into app assessment. As we know that some commercial apps are complex and hard to use by the patient group they were intended for, we have valued this section highly. Effectiveness is often mentioned in many assessment systems, but the promised effects are also dependent on the dose of the app and how intensively it is used. Patients need to consider this time constraint when deciding to make a purchase. Patients also want to know not just how effective it is, but also whether anyone does not receive any benefit. This is also important to clinicians, as patients who receive no benefit may view themselves as hopeless cases and not, as in the BlueIce exemplar, just part of the quarter of patients who report no advantage from following the app.

Our approach has, therefore, been to provide information to patients at the point of the download that allows them to make an informed decision, and which they can refer to later as part of their self-management plan.

## Responsibility in the Health App Marketplaces

If these simple T4T principles are followed, then we will have gone some way toward protecting patients. Whose job is it to monitor the T4T principles? Our view is that formal regulation is not needed. We just need the information to allow patients (and patient groups) to make informed choices. Information that is not true can be picked up by advertising standards authorities. Recent examples of this process are the US Federal Trade Commission fining of Lumosity for deceiving consumers with unfounded claims about cognitive benefits [67] and Carrot Neurotechnology for claiming that their app, Ultimeyes, can improve users’ vision [68]. Health apps are not a passing fad, and the low barrier of entry into current app marketplaces has resulted in an environment that at best confuses and at worst delays effective treatment. The problems have been highlighted but rarely have clear solutions like ours been proposed. Developers may be encouraged to produce these answers by commercial advantages, as apps with T4T principles might increase consumer comfort and produce unique revenue streams through increased adoption, not only from direct-to-patient markets, but also from health systems. They will also enjoy increased legitimacy among patient groups.

We also note that the contributions of these principles are that they are a small, yet informative, set of questions that could be adopted relatively simply. We suggest these principles as the first, but important step. Further steps could attempt to explore if these principles could be defined with more structure. However, this structure would likely require further empirical work and coordination between different stakeholders in the health app space, particularly, developers and purveyors.

Confidence in the efficacy and safety of these health apps is the least that patients should expect in making a choice to buy or use them. It is now time that existing commercial app stores, specifically the Google Play and Apple iTunes stores, step back from their libertarian ideology and adopt some rules for health app marketing. They should tighten up the definition of health apps and adopt a system, ours hopefully, to allow patients to understand what to expect from a health app. Although some might believe that this proposal is *other worldly*, starting somewhere is important. Health app marketplaces have a duty to, and health app developers a commercial advantage, from following our suggestions – we should not need to wait for another scandal or disaster before the Google Play or Apple iTunes Stores step up to the plate and help prevent worthless products being pressed on those with health needs.

## Abbreviations

FDA: Food and Drug Administration
MARS: Mobile App Rating Scale
MHRA: Medicines and Healthcare Products Regulatory Agency
NICE: National Institute of Health and Clinical Excellence
NIHR: National Institute for Health Research
T4T: Transparency for Trust
